# The fission yeast MTREC and EJC orthologs ensure the maturation of meiotic transcripts during meiosis

**DOI:** 10.1261/rna.055608.115

**Published:** 2016-09

**Authors:** Bahjat Fadi Marayati, Victoria Hoskins, Robert W. Boger, James F. Tucker, Emily S. Fishman, Andrew S. Bray, Ke Zhang

**Affiliations:** 1 Department of Biology and Center for Molecular Communication and Signaling, Wake Forest University, Winston-Salem, North Carolina 27106, USA; 2 Program of Human Genetics, Johns Hopkins School of Medicine, Baltimore, Maryland 21205, USA

**Keywords:** MTREC, elF4AIII, MAGO, meiosis, splicing, exon junction complex

## Abstract

Meiosis is a highly regulated process by which genetic information is transmitted through sexual reproduction. It encompasses unique mechanisms that do not occur in vegetative cells, producing a distinct, well-regulated meiotic transcriptome. During vegetative growth, many meiotic genes are constitutively transcribed, but most of the resulting mRNAs are rapidly eliminated by the Mmi1-MTREC (Mtl1-Red1 core) complex. While Mmi1-MTREC targets premature meiotic RNAs for degradation by the nuclear 3′–5′ exoribonuclease exosome during mitotic growth, its role in meiotic gene expression during meiosis is not known. Here, we report that Red5, an essential MTREC component, interacts with pFal1, an ortholog of eukaryotic translation initiation factor eIF4aIII in the fission yeast *Schizosaccharomyces pombe*. In mammals, together with MAGO (Mnh1), Rnps1, and Y14, elF4AIII (pFal1) forms the core of the exon junction complex (EJC), which is essential for transcriptional surveillance and localization of mature mRNAs. In fission yeast, two EJC orthologs, pFal1 and Mnh1, are functionally connected with MTREC, specifically in the process of meiotic gene expression during meiosis. Although pFal1 interacts with Mnh1, Y14, and Rnps1, its association with Mnh1 is not disrupted upon loss of Y14 or Rnps1. Mutations of Red1, Red5, pFal1, or Mnh1 produce severe meiotic defects; the abundance of meiotic transcripts during meiosis decreases; and mRNA maturation processes such as splicing are impaired. Since studying meiosis in mammalian germline cells is difficult, our findings in fission yeast may help to define the general mechanisms involved in accurate meiotic gene expression in higher eukaryotes.

## INTRODUCTION

Proper meiosis ensures that sexually reproducing organisms accurately transmit genetic information to the next generation. While nearly all diploid eukaryotic cells contain the genetic information required to undergo meiosis, only a few very specialized cells or cells exposed to particular environmental stimuli will enter meiosis, suggesting that the switch from mitosis to meiosis is precisely regulated by distinct transcriptional programs. Indeed, transcriptome analysis revealed that a massive transcriptional shift is required to enter the meiotic phase (Mata et al. 2002; Harigaya and Yamamoto 2007). In the fission yeast *Schizosaccharomyces pombe* (*S. pombe*), expression of ∼25% of the genome increases at least fourfold during meiosis (Mata et al. 2002; Chalmel et al. 2007).

During mitotic growth, most meiotic genes are silenced. Aberrant expression of meiotic genes results in cell-cycle defects in yeast and is a hallmark of some human tumors (Harigaya and Yamamoto 2007; Fratta et al. 2011). In fact, many cancers are detected by the presence of meiotic proteins. For example, the cancer/testis (CT) antigens represent a large family of meiotic proteins that are normally expressed in the spermatocytes of the testis but are often detected in cancerous tumors as well (Simpson et al. 2005). Hence, ensuring that meiotic genes remain silenced during vegetative growth is important. In fission yeast, many meiotic genes are constitutively transcribed during both vegetative growth and meiosis (Chen et al. 2011). However, in vegetative cells, their transcripts are rapidly eliminated (Harigaya et al. 2006; Harigaya and Yamamoto 2007). The best understood mechanism that selectively eliminates the meiotic transcripts during vegetative growth is the Mmi1 pathway (Harigaya et al. 2006; Chen et al. 2011). This pathway specifically targets transcripts containing the determinant of selective removal (DSR) sequence, which consists of hexameric U(U/C)AAAC repeats (Yamashita et al. 2012). Mmi1 is a sequence-dependent RNA-binding protein (Chen et al. 2011) that binds DSR-containing transcripts. Biochemical analysis has shown that Mmi1 associates with Erh1 protein to form EMC (Mmi1-Erh1 complex) (Sugiyama et al. 2016), and recruits the Red1-Mtl1-containing MTREC complex (Lee et al. 2013; Egan et al. 2014). MTREC subsequently recruits the exosome, a 3′–5′ exoribonuclease complex, to degrade these Mmi1-associated meiotic mRNAs in the nucleus during vegetative growth (Lee et al. 2013; Egan et al. 2014; Zhou et al. 2015).

The exosome is an evolutionarily conserved protein complex that functions as one of the main RNA-degradation systems in eukaryotes (Houseley et al. 2006). RNA elimination by the exosome requires 3′-processing factors including the poly(A) polymerase Pla1 and the poly(A)-binding protein Pab2, both of which are associated with MTREC (St-Andre et al. 2010; Yamamoto 2010; Egan et al. 2014). Red5, which was found to interact with Pab2 and function in RNA degradation (Sugiyama et al. 2013), copurified with MTREC in another study, and appears to be one of its core subunits (Egan et al. 2014; Zhou et al. 2015). Red5 and the other MTREC subunits Red1, Mtl1, and Iss10 are all required for degradative function (Egan et al. 2014; Zhou et al. 2015). MTREC is the *S. pombe* ortholog of the human NEXT complex (Lee et al. 2013; Zhou et al. 2015), suggesting a similar conserved mechanism for RNA degradation between *S. pombe* and higher eukaryotes (Lubas et al. 2011; Andersen et al. 2013). In addition to MTREC-associated RNA degradation, Mmi1 restricts splicing and RNA 3′ processing during mitotic growth (Chen et al. 2011). In cells with functional Mmi1-DSR degradation systems, meiotic RNAs remain unspliced and unpolyadenylated during vegetative growth, leading to their rapid degradation (Chen et al. 2011). During meiosis, or in mutants lacking Mmi1, meiotic transcripts are stabilized and are processed into fully mature mRNAs (Chen et al. 2011).

Similar to RNA polymerase II (RNAP II) mitotic transcripts, meiotic mRNAs are processed from longer precursors and packed into mRNA/protein particles (mRNPs) before being transported to the cytoplasm for translation. mRNP formation requires 5′ capping, splicing, polyadenylation at the 3′ end, and loading of mRNA packaging and export factors. These processes are precisely regulated, which may allow for multiple gene products such as the alternative splicing common in metazoans (Maniatis and Tasic 2002). In fission yeast, although ∼47% of genes contain introns, no evidence of alternative splicing that results in a single gene coding for multiple proteins has been found (Wood et al. 2002; Rhind et al. 2011). It was believed that regulatory splicing in fission yeast is limited to splice versus do-not-splice determinations (Engebrecht et al. 1991; Chen et al. 2011). About 10% of known meiotic transcripts contain introns, are spliced only during meiosis, and remain unspliced during vegetative growth (Averbeck et al. 2005). In addition, their splicing is concurrent with 3′ end maturation, and polyadenylated RNAs are not detected during vegetative growth (Potter et al. 2012). However, the exact mechanism of how specific splicing events are determined and controlled to enable meiotic transcripts to mature remains unknown (Wilhelm et al. 2008).

Mmi1-DSR-mediated degradation is inactivated during meiosis, thus Mmi1-regulated meiotic genes are efficiently expressed (Yamamoto 2010). This process relies on the coordinated action of the long noncoding RNA meiRNA and the protein Mei2 (Watanabe and Yamamoto 1994; Yamamoto 2010). During meiosis, Mei2 is phosphorylated and binds to meiRNA, forming a complex that can efficiently sequester Mmi1 from binding to DSR-containing transcripts, thereby inactivating MTREC-mediated degradation (Watanabe and Yamamoto 1994; Harigaya et al. 2006; Ding et al. 2012). During vegetative growth, Mmi1 colocalizes with Red1, a core subunit of MTREC. While Mmi1 is sequestered by meiRNA during meiosis, Red1 does not colocalize with meiRNA foci (Mei2 dot) during meiotic sequestration (Sugiyama and Sugioka-Sugiyama 2011), calling into question the function of MTREC during meiosis.

Here we show that Spac1f5.10 (pFal1, the *S. pombe* translation-initiation factor Four A Like and ortholog of eIF4AIII) interacts with the MTREC subunit Red5 to regulate the crucial splicing of meiotic transcripts during meiosis. pFal1 is a predicted Asp-Glu-Ala-Asp (DEAD)-box ATP-dependent RNA helicase (Li et al. 1999; Caruthers et al. 2000). eIF4A family proteins contain prototypical DEAD-box domains (Li et al. 1999; Lu et al. 2014). Of the three major isoforms in yeast and mammals, eIF4AI and eIF4AII are found primarily in the cytoplasm and function similarly in translational initiation (Tuteja et al. 2008; Lu et al. 2014). In contrast, eIF4AIII is a third, functionally distinct eIF4A protein that localizes mainly to the nucleus (Li et al. 1999). Like all DEAD-box family proteins, eIF4AIII exhibits nucleic-acid binding activity (Cordin et al. 2006). In mammals, eIF4AIII is a member of the exon junction complex (EJC), acting as a molecular clamp mediated by its sequence-independent nucleic-acid binding activity (Shibuya et al. 2004). It remains associated with RNA after splicing to serve as a binding platform for the other EJC proteins (Shibuya et al. 2004). Essentially, its stable binding works as a placeholder, marking RNA species that have completed their splicing (Le Hir et al. 2001; Chan et al. 2004). Constitutive binding of the EJC to RNA molecules enables recruitment of other proteins for various downstream RNA metabolic processes (Shibuya et al. 2004; Lu et al. 2014). *S. pombe* contains putative orthologs of the EJC components eIF4aIII, MAGO, Y14, and RNPS1, but lacks MLN51 (Wen and Brogna 2010).

We demonstrate that accurate meiotic gene expression during meiosis requires EJC orthologs pFal1 and Mnh1, and MTREC members Red5 and Red1. Loss of any of these proteins causes defective sporulation in diploid cells accompanied by an impairment of meiotic gene splicing. Red5 interacts with pFal1, which is associated with the other *S. pombe* orthologs of EJC, including Mnh1, Y14, and Rnps1. Unlike mammalian EJC, which cannot be assembled without Y14, we found that in *S. pombe*, the interaction between pFal1 and Mnh1 remains even in the absence of Y14 or Rnps1. Although additional evidence is necessary to fully address whether these EJC orthologs form an EJC-like complex in fission yeast, our findings pinpoint a cooperative role of elF4aIII, MAGO, MTREC, and the exosome in regulating the maturation of meiotic transcripts when meiosis is induced.

## RESULTS

### Cells without pFal1 remain viable, but display severe growth defects

In budding yeast, the eIF4AIII ortholog Fal1 is an essential protein with reported roles in rRNA processing (Kressler et al. 1997; Alexandrov et al. 2011). In *S. pombe*, prior to this study, at least one attempt to delete *spac1f5.10*^+^ (*S. pombe* Fal1, pFal1) failed (Wen and Brogna 2010), leading the authors to assume that pFal1 is essential for cell viability like its orthologs in *Drosophila melanogaster* and *S. cerevisiae* (Kressler et al. 1997; Palacios et al. 2004). In contrast, a genome-wide deletion analysis obtained viable *pfal1*Δ cells (Kim et al. 2010; Hayles et al. 2013). The strain library is commercially available, although we were not able to confirm the deletion of *pfal1*^+^ from it using standard genotyping methods. Therefore, the precise function of pFal1 in *S. pombe* remains obscure.

**FIGURE 1.**
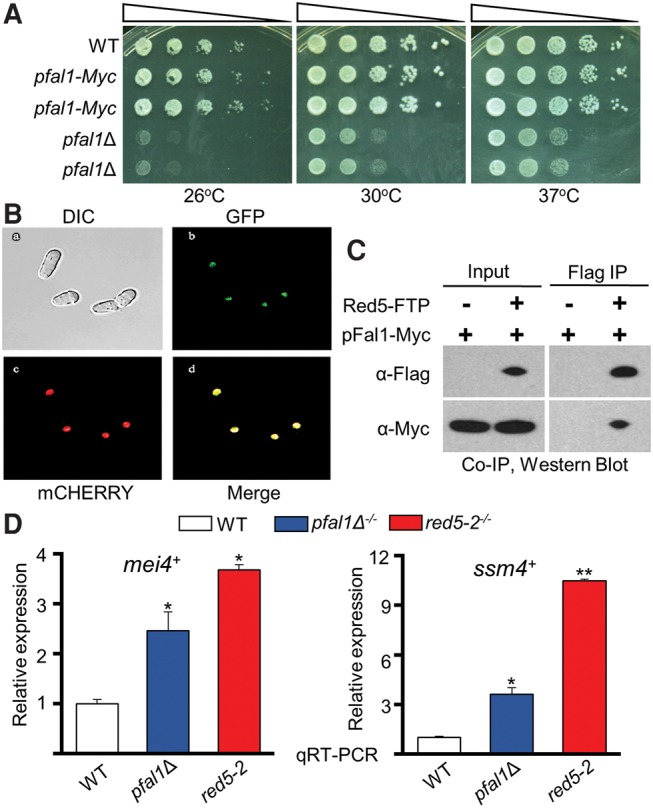
pFal1 localizes to a chromatin-rich region in the nucleus and interacts with Red5. (*A*) *pfal1*Δ haploid cells have a severe growth defect. Cells were serially diluted 10-fold, plated on rich media (YEA), and incubated at the indicated temperatures for 3 d. (*B*) pFal1-GFP colocalizes with Hta1-mCherry in confocal images of live *pombe* cells. (*C*) pFal1 interacts with Red5 as determined by Flag Co-IP and Western blot using antibodies as indicated. Immunoprecipitated samples were treated with 20 µg of RNase A/H mixture and 33 µg of DNase I before the final wash. (*D*) qRT-PCR analysis shows increased *mei4*^+^ and *ssm4*^+^ transcripts relative to *act1*^+^ normalized to wild type (WT = 1), during vegetative growth in diploid cells containing *red5-2* or *pfal1*Δ. Asterisks denote a significant difference comparing each sample with wild type, (*) *P* ≤ 0.05 and (**) *P* ≤ 0.005.

To study the role of pFal1, we generated *pfal1*Δ cells by replacing the entire open-reading frame of *pfal1*^+^ with an antibiotic-selectable marker through homologous recombination. To assay growth, cells were serially diluted 10-fold, plated on rich media (YEA), and incubated at different temperatures for 3 d. While viable, *pfal1*Δ showed a strong growth defect at all three temperatures tested (Fig. 1A). This phenotype is distinct from the deletion of budding yeast Fal1, which is lethal, suggesting a possible divergence in function between the two orthologs.

### pFal1 localizes to a distinct nuclear compartment

In *S. cerevisiae*, Fal1 primarily localizes in the nucleolus where it is required for the maturation of 40S ribosomal RNA (Kressler et al. 1997). To analyze the cellular localization of pFal1 in *S. pombe*, we created a green fluorescent protein (GFP)-tagged allele of *pfal1*^+^. This allele was combined with a mCHERRY-tagged allele of the histone protein *hta1*^+^. Visualization of a fluorescently marked histone protein allows the detection of chromatin-containing nuclear compartments. When imaged via confocal microscopy, pFal1-GFP colocalized with Hta1-mCHERRY (Fig. 1B), indicating that in *S. pombe*, pFal1 localizes to chromatin-containing regions of the nucleus and is not restricted to the nucleolus. The viability of *pfal1*Δ cells and its different pattern of nuclear localization compared to Fal1 further indicate that pFal1 may play roles distinct from those of Fal1.

### pFal1 interacts with Red5

pFal1 has previously been implicated in association with Red5, a component of the MTREC complex involved in meiotic mRNA elimination, although the function of pFal1 was not studied (Egan et al. 2014; Zhou et al. 2015). We confirmed this interaction using coimmunoprecipitation (Co-IP) followed by Western blot (Fig. 1C). To detect pFal1 by Western blot using a commercially available antibody, we added a Myc epitope tag to the carboxyl terminus of pFal1 to generate a *pfal1-Myc* allele. Unlike *pfal1Δ,* the *pfal1-Myc* allele is fully functional, and cells carrying pFal1-Myc have no growth defect at any temperatures examined by 10-fold serial dilution assay on rich media (Fig. 1A). The *pfal1-Myc* allele was further combined with a tagged Red5 allele, *red5-FTP*. FTP is a tandem affinity epitope tag containing 3× Flag and a protein A motif, separated by a Tobacco Etch Virus (TEV) protease cleavage site. We performed Co-IP by precipitating Red5-FTP with IgG-sepharose followed by Western blot using anti-Myc antibody. Interaction of Red5 and pFal1 was confirmed as pFal1-Myc and was detected in the Red5-FTP precipitated sample, but not in the mock control (Fig. 1C). Notably, the interaction was not sensitive to RNase/DNase treatment, indicating that it is mediated through protein–protein association.

### Loss of pFal1 impairs elimination of meiotic transcripts during vegetative growth

Red5 is a subunit of the MTREC complex that targets DSR-containing meiotic transcripts for degradation during mitotic growth (Lee et al. 2013; Egan et al. 2014). Red5 is essential. Conditional loss of function of Red5 (*red5-2*) reduces the efficacy of the Mmi1-DSR system, resulting in the accumulation of meiosis-specific transcripts during vegetative growth at restrictive conditions (Sugiyama et al. 2013; Egan et al. 2014). The interaction between pFal1 and Red5 indicates that pFal1 may also contribute to meiotic gene elimination during vegetative growth. To test this possibility, we compared the abundance of meiotic transcripts in *pfal1*Δ cells with that of *red5-2*, the same allele used in previous studies (Sugiyama et al. 2013). We chose to study *mei4*^+^ and *ssm4*^+^ transcripts because they both contain a DSR region (Chen et al. 2011). These transcripts are known targets of Mmi1 binding and are subsequently degraded during vegetative growth (Yamashita et al. 2012). In agreement with previous findings, we observed an increase in *mei4*^+^ and *ssm4*^+^ transcripts during vegetative growth in cells containing *red5-2* (Fig. 1D). Cells without *pfal1* accumulate intermediate levels of these transcripts between wild type and *red5-2* (Fig. 1D). These results suggest that pFal1 plays a role in the degradation of *mei4*^+^ and *ssm4*^+^ during vegetative growth, although the effect is less pronounced than that of *red5-2*.

### Meiotic defect(s) of *pfal1* mutants

Much attention has been focused on the function of MTREC/Red5 during vegetative growth, in which the complex works together with Mmi1 to facilitate the degradation and silencing of meiotic transcripts (Lee et al. 2013; Egan et al. 2014). During meiosis/sporulation, Mmi1 is sequestered by Mei2-meiRNA so that meiotic RNAs are shielded from Mmi1-mediated RNA degradation and are thereby stabilized and translated into functional proteins important for the progression of meiosis (Yamamoto 2010). It has been shown that Red1, the core component of MTREC, does not colocalize with Mmi1 during meiosis, suggesting that MTREC may possess functions distinct from its Mmi1-DSR-associated role in meiosis (Sugiyama and Sugioka-Sugiyama 2011). In addition, defective MTREC, such as that of the *red5* mutant*,* reduces sporulation efficiency, suggesting an essential role for MTREC in meiosis (Sugiyama et al. 2013). Yet, the exact roles of MTREC during this process have not been explored.

Since pFal1 interacts with Red5, we next sought to determine whether *pfal1* mutants also exhibit a sporulation defect. Diploid cells are advantageous because they allow direct analysis of meiosis independent of the mating and conjugation steps. To induce meiosis, diploid cells were first grown in rich media to mid-log phase, then transferred to SPA sporulation media and grown for an additional 24 h before being imaged. The efficiency of meiosis was then measured by analysis of the sporulation profile.

**FIGURE 2.**
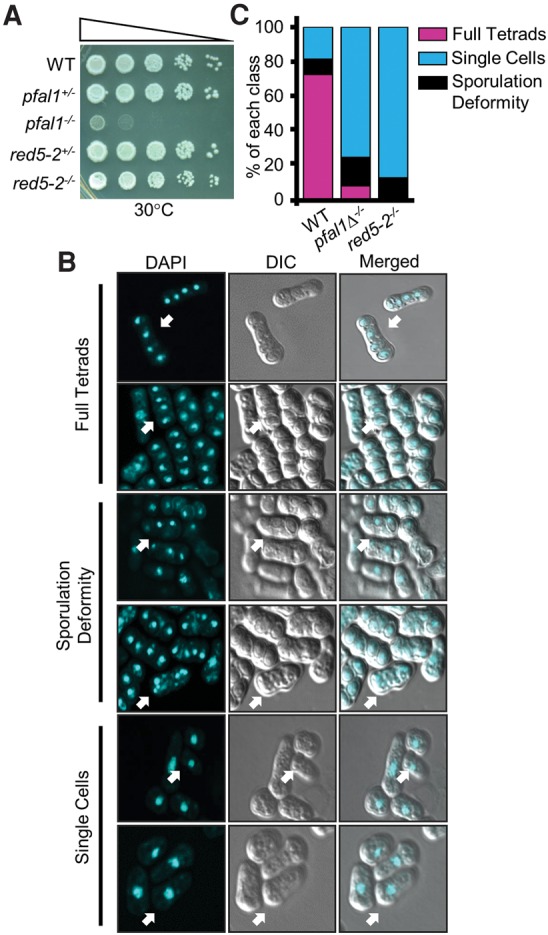
Loss of pFal1 results in defective sporulation*.* (*A*) Diploid *pfal1*Δ^−/−^ cells show a growth defect similar to that in haploid *pfal1*Δ cells. Cells were serially diluted 10-fold, plated on rich media (YEA), and incubated at 30°C for 3 d. (*B*) Sporulated cells were visualized by DAPI staining and confocal microscopy. Images are presented in three classes: full tetrads (four nuclei), deformed tetrads (three or more than four nuclei), and single cells (one nucleus). Arrowheads indicate representative cells in each class. (*C*) Imaged cells with indicated genotypes were counted and categorized as defined in *B*. Bars represent the percent of each class versus the total cells.

More than 70% of wild-type diploid cells efficiently sporulate, producing tetrads of haploid spores contained in asci following meiotic induction. Diploid *red5-2* cells show severe sporulation defects, with <1% of cells producing asci. Diploid *pfal1*Δ^−/−^ cells show a decreased sporulation efficiency compared to wild-type cells (Fig. 2B). Less than 5% of *pfal1*Δ^−/−^ cells sporulated, and ∼20% form misshapen asci on SPA medium (Fig. 2C), suggesting a meiotic defect.

### Defective splicing of meiotic genes in *pfal1* and *red5* mutants

**FIGURE 3.**
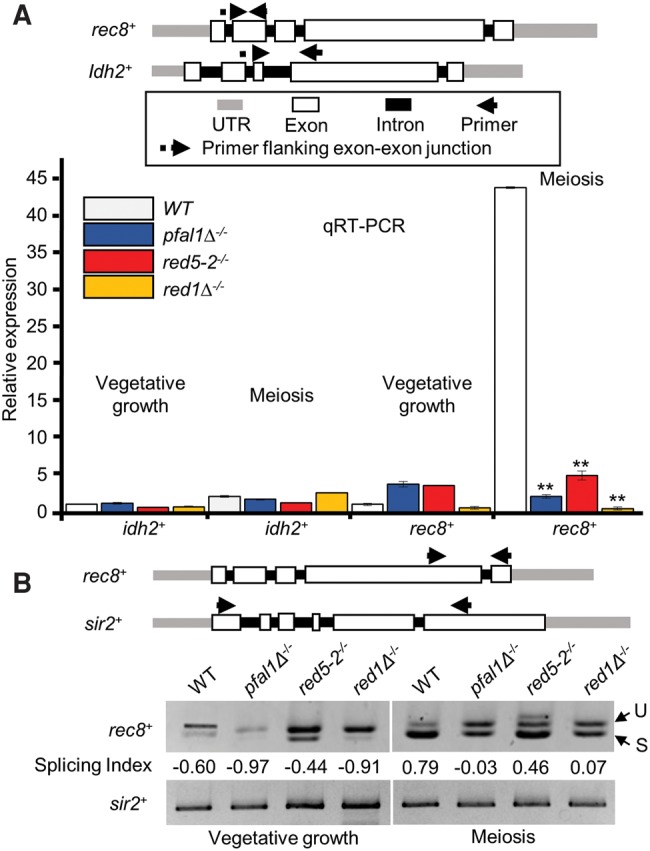
Both pFal1 and Red5 are essential for meiotic splicing of *rec8*^+^ transcripts. (*A*) qRT-PCR using primers flanking the exon–exon junction of an early meiotic gene (*rec8*^+^) and a nonmeiotic gene (*idh2*^+^) of indicated strains during mitosis (vegetative growth) or meiosis. Expression was normalized against *act1*^+^ and reported relative to vegetative wild-type values (WT = 1). (*B*) Semiquantitative RT-PCR with primers spanning two adjacent exons of *rec8*^+^ or multiple introns of *sir2*^+^. The ratios of spliced to unspliced forms of *rec8*^+^ transcripts were calculated using the splicing index. (U) Unspliced, (S) spliced.

To investigate the molecular basis behind the meiotic defect in diploid *pfal1*Δ and *red5-2* cells, we assayed the abundance and splicing patterns of meiotic transcripts during meiosis, when these genes are expressed at high levels. Since MTREC is involved in the recognition and degradation of unspliced or abnormally spliced RNAs during vegetative growth (Zhou et al. 2015), and pFal1 is the *S. pombe* homolog of eIF4aIII, a member of the EJC in mammals with a putative role in abnormal splicing-mediated decay (Shibuya et al. 2004), we analyzed meiotic transcripts of *rec8*^+^. Rec8 is a component of the meiotic cohesin complex and involved in linear element formation, chromosome pairing, and sister-chromatid cohesion during meiosis (Molnar et al. 1995). *rec8*^+^ is an early meiotic gene that is barely spliced and ultimately degraded by the exosome during vegetative growth, but is known to be regulated by splicing during meiosis (Averbeck et al. 2005). To assay the spliced RNAs of *rec8*^+^ during meiosis by qRT-PCR, we designed one primer of each pair to span an exon–exon junction so only the cDNA produced from spliced RNA species would be analyzed (Fig. 3A, top). qRT-PCR results show a large increase in *rec8*^+^ transcript levels during meiosis in wild-type cells, but no increase in *pfal1*Δ and *red5-2* mutants (Fig. 3A). To determine whether this phenotype was specific to meiotic genes, we included a constitutively spliced gene, *idh2*^+^, as a control. *idh2*^+^ codes for isocitrate dehydrogenase (NAD^+^) subunit 2, and is constitutively expressed in vegetative and meiotic cells (Marguerat et al. 2012). As expected, the relative abundance of the *idh2*^+^ transcript is not increased in meiotic diploid *pfal1*Δ, *red5-2*, or *red1*Δ cells (Fig. 3A). These results demonstrate that pFal1, Red5, and Red1 are required to generate spliced *rec8*^+^ mRNA during starvation-induced meiosis.

Next, we wanted to determine whether the decrease in meiotic transcripts observed in *pfal1*Δ, *red5-2,* and *red1*Δ mutants was specific to meiosis, or whether these genes were affected during vegetative growth as well. As part of the MTREC complex, Red5 helps to degrade DSR-containing meiotic genes during vegetative growth. Since *rec8*^+^ RNA levels are affected by mutation of *mmi1* (Averbeck et al. 2005; Chen et al. 2011), we investigated whether partial loss of Red5 function or complete loss of Red1 would result in the aberrant accumulation of *rec8*^+^ during vegetative growth. qRT- PCR results show that the loss of *pfal1*^+^ or *red5*^+^ moderately affected the level of transcript in *rec8*^+^ during vegetative growth (Fig. 3A), indicating that *rec8*^+^ is regulated by the Mmi1-MTREC system as suggested previously (Chen et al. 2011). The RNA level of the nonmeiotic, intron-containing gene *idh2*^+^ was not affected in wild-type, *pfal1*Δ, or *red5-2* mutant cells. These results show that the decreased abundance of spliced *rec8*^+^ transcripts in *pfal1*Δ or *red5-2* mutants relative to wild type is specific to meiosis, suggesting a meiotic role in regulatory splicing for pFal1 and Red5.

To further investigate this meiotic role of pFal1 and Red5, we assayed the amount of variant *rec8*^+^ transcripts, including unspliced and spliced isoforms, by semiquantitative RT-PCR with primers spanning two adjacent introns of *rec8*^+^. A nonmeiotic intron-containing gene, *sir2*^+^, is used as a control for total RNA. Like *idh2*^+^, *sir2*^+^ is constitutively expressed in both vegetative and in meiotic cells (Alper et al. 2013). Although we performed RT-PCR using the primers flanking the multiple introns of *sir2*^+^, we consistently detected only one band corresponding to the completely spliced form of *sir2*^+^ mRNA, suggesting that efficient splicing of the transcript occurs in each mutant tested. In contrast, we could clearly visualize the spliced versus unspliced form of *rec8*^+^ transcripts (Fig. 3B). To quantitatively evaluate the splicing efficiency, we calculated the splicing index, which is defined by band intensity [(spliced − unspliced)/(spliced + unspliced)]. The value, ranging from −1 (fully unspliced) to 1 (fully spliced), is normalized to total cDNA of each sample. During vegetative growth, all *rec8*^+^ transcripts show a negative splicing index, indicating that they are not spliced efficiently in both wild-type and mutant cells during mitosis (Fig. 3B). When meiosis is induced, however, *rec8*^+^ transcripts from wild-type cells show a splicing index value of 0.79, indicating a shift toward active splicing. Consistent with the meiotic defects, this shift did not occur efficiently in diploid homozygous *pfal1* and *red1* mutants, reflected by a decreased splicing index (Fig. 3). Partial loss of function of Red5 (*red5-2*) also exhibits a moderate reduction of the splicing index compared to wild-type cells during meiosis. These data indicate that pFal1, Red5, and Red1 are required for the efficient splicing of meiotic genes during meiosis.

### pFal1 interacts with the other *S. pombe* EJC orthologs

**FIGURE 4.**
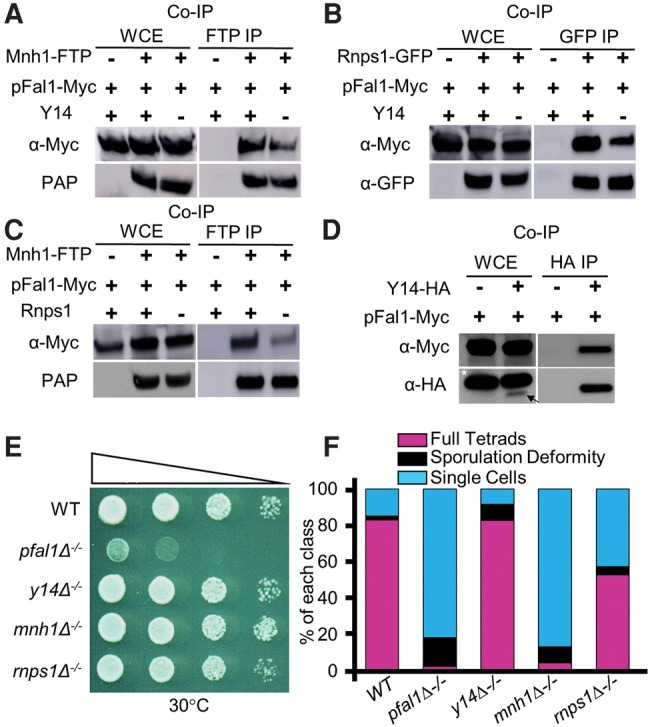
While the other EJC orthologs interact with pFal1, only Mnh1 shows a meiotic defect. (*A*) pFal1 interacts with Mnh1 with or without Y14. (*B*) pFal1 interacts with Rnps1 with or without Y14. (*C*) pFal1 interacts with Mnh1 with or without Rnps1. (*D*) pFal1 interacts with Y14. All Co-IPs were performed simultaneously using the appropriate antibodies as indicated. (*) Nonspecific binding background bands. Arrow indicates desired Y14-HA product. (*E*) Diploid cells with indicated genotypes were diluted 10-fold, spotted on rich medium, and grown at 30°C for 3 d. (*F*) Loss of either pFal1 or Mnh1 is associated with decreased sporulation efficiency, as calculated in 2C.

The roles of pFal1 and Red5 in the splicing of meiotic genes during meiosis prompted us to study the roles of their associated proteins. pFal1 is the ortholog of eIF4AIII, which is a core component of the EJC in *Drosophila* and mammals (Tange et al. 2004). In addition to pFal1, *S. pombe* orthologs of EJC core members were identified, including Y14, MAGO (Mnh1), and RNPS1 (systematic IDs SPAC23A1.09, SPBC3B9.08c, and SPBC13G1.14c, respectively). These orthologs are not required for splicing-dependent nonsense-mediated decay (NMD) (Wen and Brogna 2010), raising the question whether a mammalian-like EJC is assembled in *S. pombe*. To study this question, we generated strains expressing functional epitope-tagged Mnh1, Y14, or Rnps1, and investigated their interactions by Co-IP (Fig. 4A–D). We can easily detect interaction between pFal1-Myc and Mnh1-FTP (Fig. 4A). We also observed interactions of pFal1-Myc with Rnps1-GFP and Y14-HA (Fig. 4B,D). Notably, the interaction between pFal1-Myc and Mnh1-FTP is preserved in the absence of Y14 or Rnps1 (Fig. 4A,C). In addition, pFal1-Myc coimmunoprecipitated with Rnps1-GFP with or without Y14 (Fig. 4B). In higher eukaryotes, MAGO and Y14 form a stable dimer, and without Y14, a mammalian-like EJC cannot be formed (Lau et al. 2003; Shi and Xu 2003). Our results suggest that fission yeast does not assemble a mammalian-like EJC, which is not surprising since fission yeast does not have the ortholog of the EJC stabilizing protein MLN51, and its Y14 lacks the well-conserved amino-terminus domain found in other organisms (Wen and Brogna 2010).

### Mnh1/MAGO is indispensable for producing functional meiotic transcripts during meiosis

Since the other core EJC orthologs are associated with pFal1, it is possible that these proteins in *S. pombe* have a meiotic defect and are also involved in meiotic mRNA splicing. To investigate this, we generated deletion mutants of Y14, Mnh1, and Rnps1. Surprisingly, cells lacking these genes showed no obvious growth defects at 30°C, unlike *pfal1*Δ^−/−^ (Fig. 4E), suggesting that pFal1 performs additional functions essential for cell growth. While deletion of *y14* or *rnps1* moderately decreases sporulation efficiency, loss of *mnh1* causes severe sporulation defects (Fig. 4F), indicating an essential role of Mnh1 in meiosis.

**FIGURE 5.**
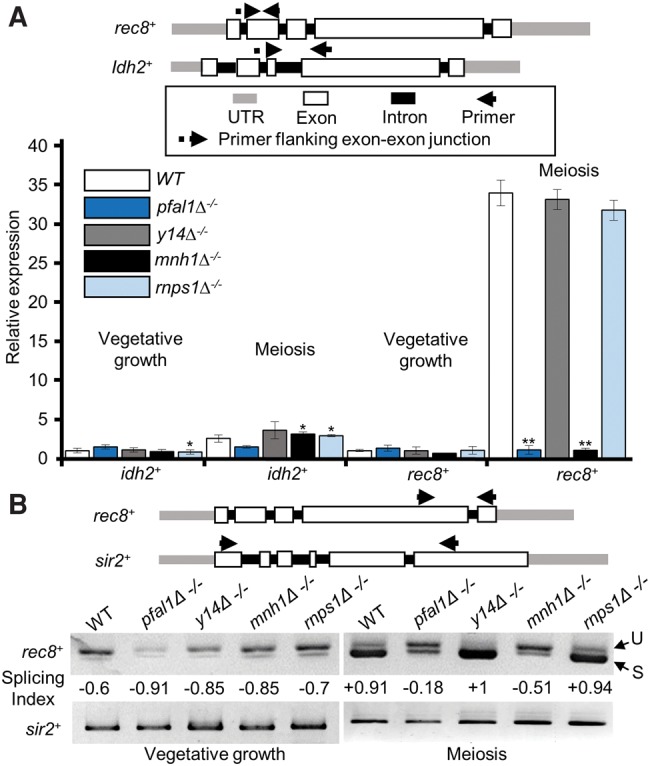
Mnh1, but not Y14 or Rnps1, is required for meiotic splicing of *rec8*^+^ transcripts. (*A*) The spliced *rec8*^+^ transcripts were analyzed by qRT-PCR using primers flanking the exon–exon junction of *rec8*^+^ and *idh2*^+^ in the indicated strains during vegetative growth or meiosis. Expression was normalized against *act1*^+^, and values reported are relative to vegetative wild type (WT = 1). (*B*) Semiquantitative RT-PCR with primers spanning two adjacent exons of *rec8*^+^ (*top*). The splicing efficiencies of *rec8*^+^ transcripts at the indicated strains were calculated using the splicing index. (U) Unspliced, (S) spliced. Asterisks denote significant difference comparing each sample with wild type within the same group (meiosis or vegetative growth), (*) *P* ≤ 0.05 and (**) *P* ≤ 0.005.

We next investigated whether Y14, Mhn1, or Rnps1 play roles in meiotic gene splicing, similar to pFal1. Using qRT-PCR, we found a reduction in spliced meiotic transcripts of *rec8*^+^ in *mnh*Δ*1*^−/−^ but not *y14*Δ^−/−^ or *rnps1*Δ^−/−^ (Fig. 5A), consistent with the degree of defective sporulation seen in these mutants. We also analyzed unspliced versus spliced transcripts of *rec8*^+^ in diploid wild-type and EJC ortholog mutants by semiquantitative RT-PCR (Fig. 5B). The splicing index of *rec8*^+^ significantly decreased in *pfal1*Δ^−/−^ and *mnh1*Δ^−/−^ homozygous diploid cells, while no reduction was observed in *y14*Δ^−/−^ and *rnps1*^−/−^ cells. These results indicate that in *S. pombe*, pFal1 and Mnh1 are required for maturation of meiotic RNAs.

### MTREC and pFal1 function in ensuring proper levels of meiotic transcripts during meiosis

**FIGURE 6.**
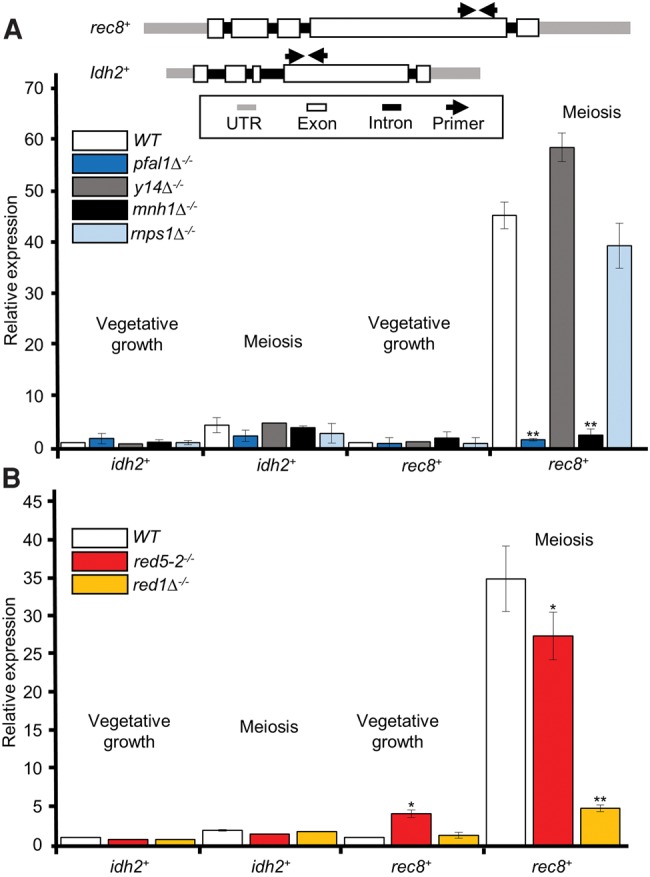
Total *rec8*^+^-transcript levels are lower in cells carrying either MTREC or EJC mutations. (*A*,*B*) Relative expression using primers within the same exon of *rec8*^+^ and *idh2*^+^ in response to deletion of EJC orthologs (*A*) or MTREC members (*B*). Expression was normalized against *act1*^+^, and values are reported relative to vegetative wild type (WT = 1). Asterisks denote significant difference comparing each sample with wild type within the same group (meiosis or vegetative growth), (*) *P* ≤ 0.05 and (**) *P* ≤ 0.005.

During meiosis, Mmi1-DSR mediated degradation must be inactivated to ensure the expression of meiotic transcripts. Although Mmi1 is sequestered by meiRNA and Mei2 (Mei2 dot), it is not clear whether MTREC has any role in the processing of meiotic transcripts, ensuring their accurate maturation and quality control. While investigating the splicing of *rec8*^+^ transcripts in meiosis, we consistently obtained fewer RT-PCR products in *pfal1*Δ^−/−^ cells (Figs. 3B, 5B). These results prompted us to investigate whether *rec8*^+^ transcripts generated in MTREC and the *pfal1* mutants might be abnormal and thereby targeted for fast elimination. To answer this question, we used primers within one exon of *rec8*^+^ or *idh2*^+^ to measure the total RNA levels by qRT-PCR in wild-type and mutant cells lacking functional MTREC or EJC orthologs. In addition to the splicing defect, the total meiotic RNA level of *rec8*^+^ was significantly lower without either functional pFal1, Mnh1 (Fig. 6A), or MTREC components (Fig. 6B). These data suggest that pFal1, Mnh1, and MTREC ensure stable expression of meiotic genes during meiosis. The decreased RNA level of *rec8*^+^ could be a result of degradation of the transcripts due to defective splicing and/or 3′ formation. It is possible that pFal1/Mnh1 and MTREC can link exosome targeting to meiotic genes through RNA-processing factors such as those involved in splicing.

## DISCUSSION

eIF4AIII has been studied primarily in mammalian tissue culture and *Drosophila*, where it functions in diverse RNA-processing pathways, including splicing, export, and degradation (Palacios et al. 2004; Shibuya et al. 2004; Malone et al. 2014). In *S. pombe,* the function of its ortholog had not been explored. In this study, we identified and investigated the function of *S. pombe* pFal1, a previously uncharacterized DEAD-box RNA helicase that plays a crucial role in the dynamic switching of meiotic gene expression during reproductive transition. We found that pFal1 is primarily localized in the chromatin-rich nuclear compartment and is required for the regulatory splicing of meiotic transcripts. Not only does it associate with MTREC via interaction with Red5, but it also associates with the other identified orthologs of EJC members Mnh1, Y14, and Rnps1. Diploid cells with defective MTREC, pFal1, or Mnh1 cannot sporulate efficiently, probably due to defective expression of meiotic transcripts, such as *rec8*^+^. Our results identify the functional coordination between MTREC, elF4aIII, and MAGO in ensuring the rapid processing and maturation of meiotic transcripts as an essential step for the proper execution of the meiotic program.

### Is there an assembled EJC in fission yeast?

In mammals, the EJC mediates splicing and nonsense-mediated decay (NMD), a translation-linked process that degrades mRNAs with premature translation termination codons (PTCs) (Shibuya et al. 2004; Le Hir and Andersen 2008). Current evidence argues against the formation of a mammalian-like EJC in fission yeast. First, open reading frames (ORFs) in fission yeast contain very few introns, so NMD probably occurs independent of splicing (Wen and Brogna 2010). Indeed, EJC orthologs are not required for NMD, and the presence of an intron in the mature transcript stimulates NMD regardless of its position relative to the PTC (Wen and Brogna 2010). Second, our study shows that EJC orthologs play distinct roles during meiosis; only pFal1 and Mnh1 exhibit meiotic defects correlated with defective splicing of meiotic transcripts (Figs. 4, 5). Third, pFal1 associates with Mnh1 without Y14 or Rnps1 (Fig. 4A,C), suggesting that pFal1 and Mnh1 form a separate complex, which interacts with MTREC. Additional biochemical analysis, such as cosedimentation, is necessary to fully address whether an EJC-like complex is formed in fission yeast.

### The potential role of the pFal1 in regulatory splicing

Transcriptome-wide analyses discovered unexpected levels of mRNA diversity, which is crucial for understanding the regulation of eukaryotic gene expression. Regulatory splicing helps to increase the complexity of mRNA isoforms, resulting in extensive proteome diversity. It can also introduce PTCs that induce RNA elimination by NMD (Kalsotra and Cooper 2011). Regulatory splicing contributes to key developmental processes such as sex determination in insects (Kalsotra and Cooper 2011), production of functionally distinct peptide hormones in mammals (Amara et al. 1982), and the transition of the meiotic developmental program in budding yeast (Engebrecht et al. 1991). In budding yeast, only 5% of genes contain at least one intron. In fission yeast, however, nearly 50% of genes contain an intron, and almost half of those contain multiple introns, indicating that splicing must be prevalent in this organism (Wood et al. 2002; Rhind et al. 2011). It was believed that regulatory splicing in both yeasts is focused on decisions of splicing versus not splicing (Engebrecht et al. 1991; Chen et al. 2011). A recent study in fission yeast suggests that mRNA levels can be regulated under different survival conditions by recruiting Mmi1 to “decay-promoting” introns (Kilchert et al. 2015). Some evidence also supports the presence of alternative splicing in fission yeast (Awan et al. 2013); however, it is not known whether functional proteins are produced as a result of exon skipping.

In this study, we found that only a very small proportion of *rec8*^+^ transcripts are spliced during vegetative growth; however, splicing is efficient and common during meiosis (Fig. 3). This observation is in agreement with a role for regulatory splicing in the switch to the meiotic developmental program. Without pFal1, *rec8*^+^ is not efficiently spliced during meiosis, resulting in a significantly different profile of band intensity compared to that of wild-type cells. In addition to a role for the pFal1 in splicing, we found that the total RNA of *rec8*^+^ decreases when pFal1 and Mnh1 are mutated (Fig. 6), suggesting that unspliced transcripts are most likely targeted for degradation by the MTREC–exosome pathway. We attempted to combine *pfal1* or *mnh1* mutant with a deletion of Rrp6 (*rrp6*Δ), the core catalytic subunit of the nuclear exosome, but recovered no double mutants, suggesting a synthetic lethality between these EJC orthologs and the exosome (data not shown). This genetic analysis implies that pFal1-Mnh1 and the exosome have overlapping functions essential for survival. It is likely that MTREC links the function of these proteins with the exosome.

### Multifaceted functions of MTREC in mitotic growth and meiosis

**FIGURE 7.**
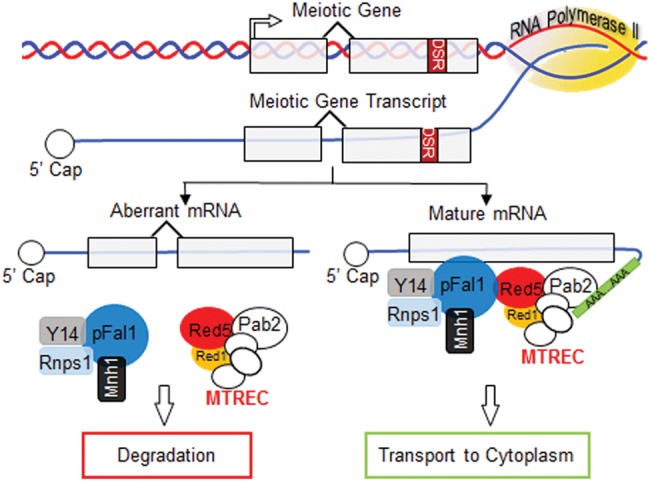
Meiotic roles for the multifaceted MTREC complex, transcript processing, and facilitation of their maturation. Abnormal meiotic transcripts generated in the absence of functional MTREC and some EJC orthologs are targeted for degradation. Physical interactions between MTREC and the EJC ensure their close proximity to accurately govern the regulation of meiotic gene expression.

To suppress meiosis in vegetative fission yeast, Mmi1 binds to DSR-containing meiotic transcripts and cooperates with MTREC to selectively degrade them (Yamanaka et al. 2010). During meiosis, relocalization of Mmi1 to the Mei2 dot allows meiotic transcripts to accumulate and be translated into meiosis-specific proteins (Yamanaka et al. 2010). The core component of MTREC, Red1, is colocalized with Mmi1 during vegetative growth but does not move with it to mei2 dot during meiosis (Sugiyama and Sugioka-Sugiyama 2011). This difference suggests that MTREC and Mmi1 are independently regulated during meiosis (Sugiyama and Sugioka-Sugiyama 2011). Although it is known that MTREC is crucial for DSR-mediated mRNA decay and interacts with splicing factors (Lee et al. 2013; Zhou et al. 2015), its exact role in meiosis was not known. Our results demonstrate its specific role in regulatory splicing of meiotic genes and support a positive role during meiosis in contrast with its role in the DSR-mediated degradation of meiotic RNAs during vegetative growth. MTREC may facilitate the maturation of meiotic transcripts by recruiting RNA-processing factors, including splicing and 3′ end formation factors. This hypothesis is supported by the observation that when *rec8*^+^ transcripts are not properly spliced during meiosis following the loss of pFal1, the abundance of *rec8*^+^ transcripts decreases (Figs. 3B, 5B, 6). Since Red5 is associated with cleavage and poly(A) machineries (Lee et al. 2013; Egan et al. 2014; Zhou et al. 2015), the protein–protein interaction between pFal1 and Red5 may provide evidence of a physical link between splicing and 3′ end processing factors (Fig. 7). The pFal1-Mnh1 may bind upstream of the exon–exon junction where it interacts with Red5 to recruit other processing factors including cleavage and poly(A) machinery. The interactions between these factors ensure their close proximity to the site of meiotic RNA transcription. Governing accurate meiotic gene expression, MTREC performs two seemingly opposite roles: DSR-mediated degradation of meiotic transcripts in mitosis and processing and maturation of meiotic transcripts in meiosis.

Meiosis is an immensely complex process that requires the concerted effort of myriad gene products (Yamanaka et al. 2010). Elucidating the processes that control the expression of meiotic genes is necessary to understand eukaryotic sexual reproduction. Considering that MTREC and the EJC orthologs are conserved from yeast to humans (Wen and Brogna 2010; Zhou et al. 2015), our results may provide insights that are broadly applicable across eukaryotes into various RNA-processing pathways essential for meiosis.

## MATERIALS AND METHODS

### Strains and cell culture

Both the Red5-FLAG (ts) and Red5-FTP strains were generously provided by Dr. Tamás Fischer, Heidelberg University Biochemistry Center. All other strains were created using standard methods described previously (Bahler et al. 1998). Standard methods of *S. pombe* culture were followed as previously described (Moreno et al. 1991; Forsburg 2003). Vegetative cells were maintained at 30°C in YEA media. Diploid cells were created by inducing conjugation of cells of complementary *h*^+^ and *Smt0* mating types. Conjugation was induced by plating the mixture of two yeast strains of opposite mating type on SPA media at 26°C for 18 h. Using strains containing the intragenically complementary *ade6-M210* and *ade6-M216* alleles allowed for selection for diploid cells on media lacking adenine (Szankasi et al. 1988). *S. pombe* cells proliferate and remain as diploids if starvation is interrupted shortly after conjugation. To this end, newly formed diploid cells were transferred into YEA media at 30°C. They grew in liquid YEA media until mid-log phase when OD at 595 nm reached 0.3–0.5. They were then harvested by centrifugation at 1000 RCF, washed with sterile double-distilled H_2_O, and resuspended in the same amount of SPA sporulation media. Around 1OD cells were taken at this point and counted as time 0. The rest of the cells were incubated in SPA sporulation media at 30°C for 6 h before harvest. A list of strains used in this study is available online: http://college.wfu.edu/biology/people/faculty/zhang/zhang-lab/zhang-lab-links/.

### Quantifying sporulation efficiency

After sporulation, 5 µL of each culture was placed on a glass coverslip and heat-fixed at ∼70°C on a ceramic hotplate. The coverslip with attached cells was placed onto a drop of DAPI mounting media (Southern Biotech) and then imaged using a confocal microscope. Sporulation efficiency was measured using the cell counter Image J plugin as previously described (Tucker et al. 2016). Cells with one or two nuclei were considered to be growing vegetatively; cells with four nuclei were considered to form a full tetrad; and cells with any other number of nuclei were considered to have a sporulation deformity. Efficiency was reported as the percentage ratio of each group versus the total counted cells.

### qRT-PCR analysis of meiotic gene expression

Total RNA was isolated from cells during vegetative growth and 6 h after placement in SPA sporulation media using the Masterpure Yeast RNA Purification Kit (Epicentre). cDNA was synthesized using M-MLV reverse transcriptase (Promega) according to the manufacturer's specifications and primed with Oligo dT^18^ (Fisher). The analysis of meiotic gene expression was performed by qPCR using primers specific to the mature mRNA. This was accomplished by designing one primer of each pair so that 3–7 bases on its 3′ end spanned an exon–exon junction to ensure that only spliced transcripts were detected. At least two biological repeats were performed for all experiments. A Student's *t*-test was performed (two-tailed distribution) in order to compare each of the indicated sample values with wild-type values of the same group (mitosis or meiosis). Error bars represent standard error (s.e.). Asterisks denote the level of significance, (*) *P* ≤ 0.05 and (**) *P* ≤ 0.005 (Figs. 1, 3A, 5A, and 6).

### Semiquantitative analysis of meiotic transcripts

Semiquantitative PCR was used to examine the abundance of spliced transcripts relative to unspliced transcripts. cDNA was amplified by PCR using primers spanning at least one intron. The resulting amplicons were separated by 1% agarose electrophoresis, stained with SYBR green at a 1:10,000 dilution in TBE, and imaged on an Amersham Imager 600 RGB (GE Healthcare). Band intensity was quantified using ImageJ software, and the splicing index of band intensity [(spliced − unspliced)/(spliced + unspliced)] was calculated.

### Coimmunoprecipitation (Co-IP) and Western blotting

Coimmunoprecipitation experiments were carried out as described previously (Zhang et al. 2008; Gerace and Moazed 2015). All strains were grown simultaneously in 150 mL cultures to an equal OD_595_ ≈ 1.5. Pelleted cells were washed with 1× PBS and 0.5 g pellets were saved. Whole-cell lysate (WCE) was obtained through bead beating (425–600 µm Glass Beads, Sigma-Aldrich) and tandem spinning followed by incubation with 2 μL Benzonase (25 unit/µL, EMD Millipore) for 2 h at 16°C. Protein A/G Agarose (Roche) coupled with the appropriate antibody were used to immunoprecipitate Myc-, HA-, and GFP-tagged proteins. FTP-tagged proteins were pulled down directly with IgG Sepharose (GE Healthcare). IP samples were incubated on an end-over-end rotator for 2 h at 4°C before being washed and eluted with 1× SDS Sample Buffer. Immunoprecipitated fractions and the equivalent of 2%–5% of the input extracts were analyzed by Western blot using anti-Flag (M2, Sigma), anti-Myc (A14, Santa Cruz), PAP (Sigma), anti-HA (Y11, Roche), or anti-GFP (Roche) antibodies.
